# Clinical phenotypes, endotypes, and biomarker profiles of immediate rituximab hypersensitivity reactions: a prospective pharmacovigilance study

**DOI:** 10.3389/falgy.2026.1903523

**Published:** 2026-09-03

**Authors:** Narapong Yotinnoratham, Wannada Laisuan, Kumutnart Chanprapaph, Silada Kanokrungsee, Chavachol Setthaudom, Supornchai Onpun, Pungjai Mongkolpathumrat, Supranee Buranapraditkun, Apinya Chungcharoenpanich, Nichapha Dechapaphapitak, Supa Oncham, Nattakirana Tongdee, Sananya Khamkaew, Ratchaya Lertnawapan, Jettanong Klaewsongkram

**Affiliations:** 1 Division of Allergy Immunology and Rheumatology, Department of Medicine, Faculty of Medicine, Ramathibodi Hospital, Mahidol University, Bangkok, Thailand; 2 Division of Dermatology, Department of Medicine, Faculty of Medicine, Ramathibodi Hospital, Mahidol University, Bangkok, Thailand; 3 Department of Dermatology, Faculty of Medicine Siriraj Hospital, Mahidol University, Bangkok, Thailand; 4 Immunology Laboratory, Department of Pathology, Faculty of Medicine Ramathibodi Hospital, Mahidol University, Bangkok, Thailand; 5 Division of Allergy and Clinical Immunology, Department of Medicine, Center of Excellence for Skin and Allergy Research, Faculty of Medicine, Chulalongkorn University, Bangkok, Thailand; 6 Department of Medicine, Faculty of Medicine, Thammasat University, Pathumthani, Thailand

**Keywords:** anaphylaxis, biologic, cytokine release syndrome, hypersensitivity, interleukin-6, mixed reaction, monoclonal antibody, phenotype

## Abstract

**Background:**

Rituximab (RTX) has been associated with immediate hypersensitivity reactions (HSRs) involving multiple phenotypes and endotypes.

**Objective:**

To characterize the clinical phenotypes and endotypes of immediate RTX HSRs.

**Methods:**

A prospective study was conducted as part of a pharmacovigilance program from July 2020 to December 2021. Participants who experienced immediate RTX HSRs were enrolled. Skin testing, basophil activation testing (BAT), IFN-*γ* ELISpot assays, and IgG anti-drug antibody (ADA) ELISA assays were performed.

**Results:**

Seventeen patients with immediate RTX reaction were enrolled, including 9 with infusion-related reactions (IRRs) and 8 with immediate RTX HSRs. Among 13 patients who underwent biomarkers testing, all 5 patients with IRRs showed negative result for all biomarkers. Among the eight patients with immediate RTX HSRs, elevated serum tryptase levels during acute reaction onset were observed in four patients, suggesting possible type I reaction, although definite classification was limited by the reaction. Two of the 8 patients demonstrated elevated IL-6 levels together with increased serum tryptase levels, suggesting mixed reactions (type I/CRS). The remaining 4 patients could not be assigned to a specific endotype and were therefore categorized as unclassified. BAT results were negative in all cases.

**Conclusion:**

Immediate RTX HSRs demonstrated heterogeneous phenotypes and endotypes. IRRs were the most common phenotype and occurred exclusively during the first RTX cycle. Skin testing, ADA detection, and serum IL-6 measurement may contribute to endotype characterization, although larger prospective studies are needed to establish their diagnostic utility.

## Introduction

Over the past two decades, monoclonal antibodies (mAbs) have become an integral component of treatment strategies for a wide range of conditions, including autoimmune diseases, dermatologic disorders, and malignancies (1). As the use of mAb therapies continues to expand because of their proven efficacy, the incidence of hypersensitivity reactions, including anaphylaxis, is expected to increase (2). Analysis of the U.S. Food and Drug Administration Adverse Event Reporting System (FAERS) database from its inception to September 2025 identified mAbs was the top 10 frequently implicated drug class associated with anaphylaxis.

Biologic agents are large, complex molecules with the potential to induce immunogenic responses, leading to the development of anti-drug antibodies (ADAs) (3). Hypersensitivity reactions (HSRs) to biologics have been classified according to their clinical phenotypes, underlying endotypes, and associated biomarkers. Immediate reaction to rituximab includes type I reactions (IgE-mediated and non-IgE-mediated), infusion-related reactions (IRRs), cytokine release syndrome (CRS), and mixed reactions involving features of both type I reactions and CRS. Non-immediate HSRs include serum sickness reactions (type III reactions) and type IV reactions, which may range from maculopapular eruptions to severe cutaneous adverse reactions (SCARs) (4, 5). Both immediate and non-immediate HSRs may further be categorized as ADA-mediated or non-ADA-mediated reactions (4).

HSRs may occur during either the first exposure to a biologic agent or following repeated administrations (6). The clinical manifestations of immediate HSRs range from mild localized cutaneous symptoms to severe, life-threatening systemic reactions. IRRs typically occur during the first infusion cycle, are generally mild to moderate in severity, and often diminish with subsequent infusions. In contrast, fever, chills, pain, headache, rigors, and nausea are characteristic manifestations of CRS. Nevertheless, substantial clinical overlap exists between type I reactions and CRS, making differentiation challenging in routine clinical practice. Biomarkers such as serum tryptase, interleukin-6 (IL-6), skin testing with the culprit biologic agent, and *in vitro* assays are valuable tools for elucidating the underlying endotype of immediate HSRs and may facilitate pathogenesis-directed management (4, 7).

Rituximab (RTX) accounts for approximately one-third of biologic-associated anaphylaxis reports in the United States (8). IRRs represent the most common immediate reactions to RTX and have been reported in up to 77% of treated patients, whereas true hypersensitivity reactions occur in approximately 5%–10% of cases (6). The clinical spectrum of RTX-induced reactions ranges from mild infusion-related symptoms to severe anaphylaxis. RTX-induced HSRs are particularly important in diseases with limited therapeutic alternatives, such as adult-onset immunodeficiency associated with anti-interferon-*γ* autoantibodies (9, 10).

Given the heterogeneity of RTX-induced immediate HSRs and the limited data regarding their underlying mechanisms, this study aimed to characterize the clinical phenotypes and endotypes of immediate RTX HSRs. To achieve this objective, we performed skin testing and a panel of *in vitro* investigations, including the basophil activation test (BAT), interferon-*γ* (IFN-*γ*) ELISpot assay, and anti-drug antibody (ADA) enzyme-linked immunosorbent assay (ELISA).

## Methods

### Patient selection

A prospective, longitudinal, single-center study was conducted as part of a pharmacovigilance program at a tertiary-care university hospital between July 2020 and December 2021. The study protocol was approved by the Ethics Committee of Ramathibodi Hospital (COA.MURA2021/151) and was conducted in accordance with the principles of the Declaration of Helsinki. Written informed consent was obtained from all participants.

Adult patients (≥18 years of age) who experienced immediate reaction to RTX were eligible for enrollment. Demographic and clinical data were collected, including age, sex, underlying diseases, history of drug allergy, RTX products (originator or biosimilar), treatment cycles, and clinical manifestations of the reactions. Reaction severity was graded according to Brown's anaphylaxis classification (11).

To characterize the underlying endotypes of immediate reaction to RTX, the following investigations were performed: skin testing with RTX, basophil activation testing (BAT), interferon-gamma (IFN-*γ*) ELISpot assay, anti-drug antibody (ADA) enzyme-linked immunosorbent assay (ELISA), serum tryptase measurement, and interleukin-6 (IL-6) determination. For patients who developed immediate reaction to RTX, acute-phase blood samples were collected during the reaction for biomarker analysis. Serum tryptase samples were obtained within 30 min of symptom onset, whereas serum IL-6 samples were collected within 1 h of symptom onset. Biomarker samples obtained outside this predefined sampling window were considered non-interpretable and excluded from biomarker-specific analysis because delayed sampling may reduce diagnostic accuracy.

Infusion-related reactions (IRRs) were defined as immediate reactions that improved with premedication and/or reduction of the infusion rate and demonstrated a self-limiting course upon subsequent exposure. Type I reactions were defined as immediate hypersensitivity reactions with clinical features consistent with mast cell and/or basophil activation, supported by evidence of either IgE-mediated or IgG-mediated mechanisms, including positive skin testing, positive BAT, elevated serum tryptase levels, or ADA positivity suggestive of antibody-mediated immune activation. CRS was defined by clinical features compatible with cytokine-mediated reactions together with elevated serum IL-6 levels. Mixed reactions were defined by the presence of features supporting both type I reactions and CRS. Delayed or non–type I immune-mediated reactions were supported by positive interferon-gamma (IFN-*γ*) ELISpot results.

### Serum tryptase

Serum tryptase levels were measured at two time points: 30 min after the onset of the reaction and at least 24 h after symptom resolution. Acute mast cell activation was assessed using the international consensus equation for serum tryptase (12).

### Skin test

Skin testing with RTX was performed at least 4 weeks after the index reaction. Recommended concentrations were used: 10 mg/mL for skin prick testing (SPT) and 0.1 mg/mL and 1 mg/mL for intradermal testing (IDT). Normal saline and histamine served as negative and positive controls, respectively. SPT and IDT were interpreted after 15–20 min using standardized criteria for immediate hypersensitivity reactions.

An SPT result was considered positive when the wheal diameter was >3 mm compared with the negative control. For IDT, a wheal diameter at least 2 mm greater than that of the negative control was considered positive (13).

### Basophil activation test

BAT was performed according to a previously published protocol (14). Flow cytometric analysis was conducted using a BD FACSCalibur^TM^ flow cytometer (Becton Dickinson, Franklin Lakes, NJ, USA) in combination with the Flow CAST® kit (Bühlmann Laboratories AG, Switzerland). Basophils were identified using CCR3 expression, and activation was assessed by CD63 upregulation.

Three milliliters of peripheral blood were collected in ethylenediaminetetraacetic acid (EDTA) tubes and processed within 4 h of collection. For each sample, 50 μL of whole blood was incubated with 100 μL of stimulation buffer containing IL-3, calcium, and heparin, together with 20 μL of anti-CCR3-FITC and anti-CD63-PE antibodies.

Four conditions were tested: negative control, positive control (anti-Fc*ε*RI antibody), RTX at 0.25 mg/mL, and RTX at 1 mg/mL. Samples were incubated for 15 min at 37 °C, followed by erythrocyte lysis, washing, and acquisition on the flow cytometer. Data were analyzed using CellQuest Pro software.

A minimum of 500 basophils was required for analysis. Basophils were identified as CCR3high/SSClow cells. Activated basophils were defined as CCR3+/CD63 + cells. BAT positivity was determined by either ≥5% activated basophils or a stimulation index (SI) ≥ 2, calculated as the ratio of activated basophils after RTX stimulation to baseline activation in the negative control.

### IFN-*γ* ELISpot assay

Peripheral blood samples were collected in acid citrate dextrose (ACD) tubes and processed within 4 h. Peripheral blood mononuclear cells (PBMCs) were isolated by Ficoll-Hypaque density-gradient centrifugation.

An IFN-*γ* ELISpot assay kit (Mabtech, Stockholm, Sweden) was used to quantify RTX-specific T-cell responses. Polyvinylidene fluoride (PVDF) membrane plates were coated overnight with anti-IFN-*γ* capture antibody (5 μg/mL) at 4 °C and subsequently blocked with R10 medium.

PBMCs (2.5 × 10⁵ cells/well) were incubated with RTX at concentrations of 5 μg/mL and 50 μg/mL for 40 h at 37 °C in 5% CO₂. Following incubation, plates were processed according to the manufacturer's protocol and analyzed using an ImmunoSpot S6 Ultimate Analyzer (Cellular Technology Limited).

Results were expressed as spot-forming cells (SFCs) per 10⁶ PBMCs after subtraction of background responses from unstimulated controls. A response exceeding 18 IFN-*γ* SFCs/10⁶ PBMCs (mean + 2 SD of controls) was considered positive (15).

### Anti-drug antibody ELISA

Anti-RTX antibodies were quantified using a commercial ELISA kit (ab237654, BioVision/Abcam, Cambridge, UK) according to the manufacturer's instructions.

Serum samples were serially diluted and analyzed together with standard calibrators and quality controls. Absorbance was measured at 450 nm using a microplate reader. Assay validity required an OD450/650 ≥ 1.000 for the high control and <0.200 for the low control.

The positivity threshold was determined as three times the mean optical density of negative controls. Samples demonstrating ≥25% inhibition in the confirmatory assay were considered ADA-positive.

### Statistical analysis

Continuous variables were summarized as median and interquartile range (IQR), whereas categorical variables were reported as frequencies and percentages. No formal hypothesis testing or inferential statistical analyses were conducted because the study was not adequately powered to detect statistically meaningful differences. Statistical analyses were performed using IBM SPSS Statistics for Windows, version 25.0 (IBM Corp., Armonk, NY, USA). A two-sided *P* value <0.05 was considered statistically significant.

## Results

### Patient characteristics and clinical presentation

During the study period (July 2020 to December 2021), 1,113 patients received RTX, of whom 17 patients who experienced immediate reactionto RTX were enrolled in this study.

Patient characteristics are summarized in Table 1. Of the 17 patients who experienced immediate reaction to RTX, 15 (88.23%) had received RTX biosimilars. The median age at reaction onset was 43 years (range, 18–68 years), and 12 patients (70.58%) were female.

**Table 1:** Patient characteristics of immediate reaction to rituximab.

Patient’s characteristic	Total, *n* = 17 (%)
Rituximab drug	
Originator	2 (11.8)
Biosimilar	15 (88.2)
Age (yrs) – Median (min, max)	43 (18, 68)
Female	12 (70.6)
Underlying diseases	
Autoimmune diseases^a^	15 (88.2)
Primary CNS Lymphoma	1 (5.9)
Anti-interferon-*γ* autoantibody	1 (5.9)
Anaphylaxis grading severity^b^	
Grade I	5 (29.4)
Grade II	8 (47.1)
Grade III	4 (23.5)
Organs involvements	
Skin and mucocuteneous	10 (58.8)
Respiratory	10 (58.8)
Cardiovascular	9 (52.9)
Gastrointestinal	2 (11.8)
Other (headache, backache)	2 (11.8)
Comorbidity	
Diabetes mellitus	3 (17.7)
Hypertension	2 (11.8)
Dyslipidemia	1 (5.9)
History of drug allergy	1 (5.9)
History of previous biologic treatment	6 (35.3)

a Autoimmune disease; Pemphigus vulgaris, IgG4 related disease, Systemic Lupus Erythematosus, Rheumatoid arthritis, Stiff person syndrome, Pemphigus foliaceus, Autoimmune inner ear disease, Autoimmune retinopathy.

b Brown anaphylaxis grading system.

Autoimmune diseases were the most common underlying conditions, accounting for 15/17 (88.23%) cases. Pemphigus vulgaris was the most frequent diagnosis (7/17, 41.17%), followed by IgG4-related disease (2/17, 11.76%). Six patients (35.29%) had previously received other biologic therapies without experiencing hypersensitivity reactions. Clinical characteristics and potential confounding factors are summarized in Supplementary Table S1. Most patients received concomitant immunosuppressive therapy and rituximab premedication including corticosteroid and anti-histamine, although corticosteroid exposure before acute biomarker sampling varied across patients.

Skin and respiratory manifestations were the most common clinical features, each occurring in 58.82% of patients. Cardiovascular involvement was observed in 52.94% of cases, whereas gastrointestinal symptoms occurred in 11.76%. According to Brown's classification, anaphylaxis grade II was the most common severity category, accounting for 47.06% of immediate reaction to RTX.

### Clinical phenotypes and endotypes

Infusion-related reactions (IRRs) were identified in 9 of 17 patients. All IRRs occurred during the first cycle of RTX administration and were classified as Brown grade I or II reactions. Eight of 17 patients were diagnosed immediate RTX HSRs.

Skin testing (SPT and IDT), basophil activation testing (BAT), IFN-*γ* ELISpot assay, and ADA ELISA were performed in 13 patients, including five patients with IRRs and eight patients with immediate RTX HSRs. All evaluated patients in the IRR group showed negative results for all tested biomarkers.

**Figure 1:**
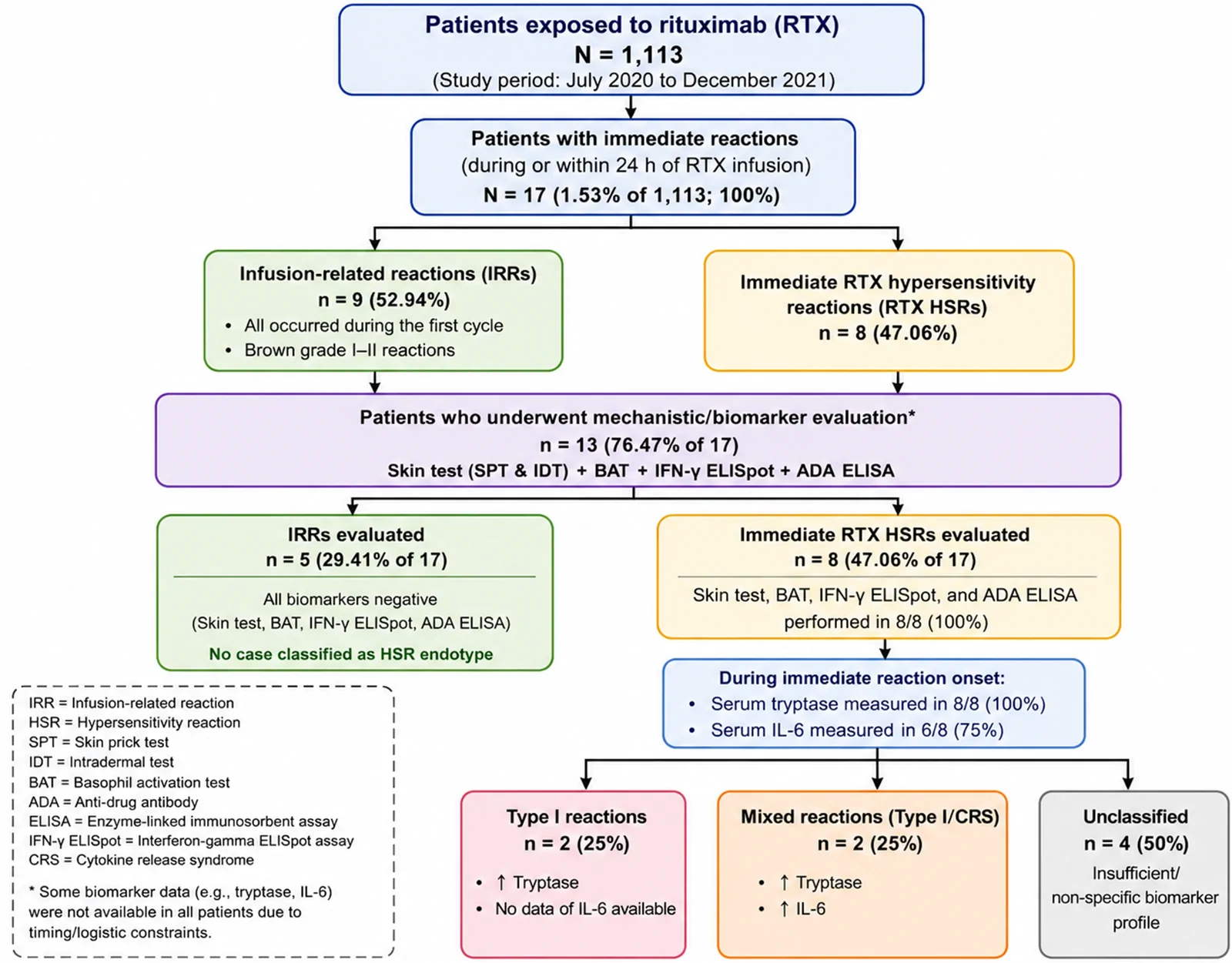
Clinical phenotypes and mechanistic evaluation of immediate rituximab reactions. Flowchart showing the identification and classification of immediate reactions among 1,113 patients exposed to rituximab (RTX) between July 2020 and December 2021. Patients with immediate reactions were categorized as infusion-related reactions (IRRs) or immediate RTX hypersensitivity reactions (HSRs). Patients undergoing mechanistic evaluation were assessed using skin testing, basophil activation testing (BAT), IFN-γ ELISpot, and anti-drug antibody (ADA) ELISA, with serum tryptase and interleukin-6 (IL-6) measured during the acute reaction when available. Immediate RTX HSRs were further classified according to the biomarker profile as type I, mixed type I/cytokine release syndrome (CRS), or unclassified reactions. Some biomarker data were unavailable because of timing or logistical constraints. RTX, rituximab; IRR, infusion-related reaction; HSR, hypersensitivity reaction; SPT, skin prick test; IDT, intradermal test; BAT, basophil activation test; ADA, anti-drug antibody; ELISA, enzyme-linked immunosorbent assay; IFN-γ, interferon-gamma; CRS, cytokine release syndrome.

Among the eight patients with immediate RTX HSRs, elevated serum tryptase levels during acute reaction onset were observed in four patients, suggesting possible type I reaction, although definite classification was limited by incomplete biomarker data. Two patients demonstrated elevated serum IL-6 levels during the reaction, together with increased serum tryptase levels, suggesting mixed reactions (type I/CRS). The remaining four patients could not be assigned to a specific endotype and were therefore categorized as unclassified (Figure 1, Table 2).

**Table 2:** Phenotypes and endotype characterized by clinical presentation, biomarkers and *in vitro* test.

Case no.	Underlying Disease	Biologic Agents	Switching RTX	Dose at which HSRs occurred	Clinical presentations	Anaphylaxis grading	Biomarkers		Skin Test	BAT	IFN-γ ELISpot	ADA ELISA
							Tryptase (ng/mL)	IL-6 (pg/mL)				
1	SLE	B	O > B	3rd	Urticaria, pruritus, angioedema, dyspnea	II	3.5	NA	Pos	Neg	Neg	Neg
2	Stiff person syndrome Severe AA CGVHD	B	O > B	3rd	Urticaria, pruritus, dyspnea, chest pain, hypoxemia	III	21.0	NA	Neg	Neg	Neg	Pos
3	Pemphigus vulgaris	B	B > B	4th	Urticaria, pruritus, angioedema, dyspnea, back pain	II	16.2	68.1	Pos	Neg	Neg	Neg
4	Anti-IFN-γ auto-Ab	B	B > B	4th	Dyspnea, chest pain, tachycardia, nausea, abdominal pain, hypoxemia, hypotension	III	10.8	43.8	Neg	Neg	Pos	Pos
5	Primary CNS Lymphoma	B	No	4th	Bradycardia,dyspnea	II	NA	NA	Neg	Neg	Neg	Neg
6	Rheumatoid arthritis	B	B > B	2nd	Pruritus, dyspnea, chest pain, tachycardia	II	5.3	NA	Neg	Neg	Neg	Neg
7	Pemphigus foliaceus	B	B > B	3rd	Urticaria, pruritus, dyspnea, chest pain, nausea, abdominal pain, hypoxemia	III	NA	NA	Neg	Neg	Neg	Neg
8	IgG4-related disease	B	No	2nd	Generalized erythema, pruritus, dyspnea, chest pain, hypoxemia	III	2.4	1.5	Neg	Neg	Neg	Neg

AA, aplastic anemia; ADA, anti-drug antibody; Anti-IFN-γ auto-Ab, anti-interferon-gamma autoantibody; B, biosimilar rituximab; BAT, basophil activation test; B > B, switch from one rituximab biosimilar to another rituximab biosimilar; CGVHD, chronic graft-versus-host disease; CNS, central nervous system; HSR, hypersensitivity reaction; IFN-γ ELISpot, interferon-gamma enzyme-linked immunospot assay; IgG4-RD, IgG4-related disease; IL-6, interleukin-6; NA, not available; Neg, negative; O > B, switch from rituximab originator to rituximab biosimilar; Pos, positive; RTX, rituximab; SLE, systemic lupus erythematosus.

Immediate RTX HSRs predominantly occurred during the third and fourth treatment cycles and were generally associated with more severe clinical manifestations, corresponding to Brown grade II or III anaphylaxis. In contrast, IRRs occurred exclusively during the first RTX cycle and were limited to grade I or II reactions.

**Figure 2:**
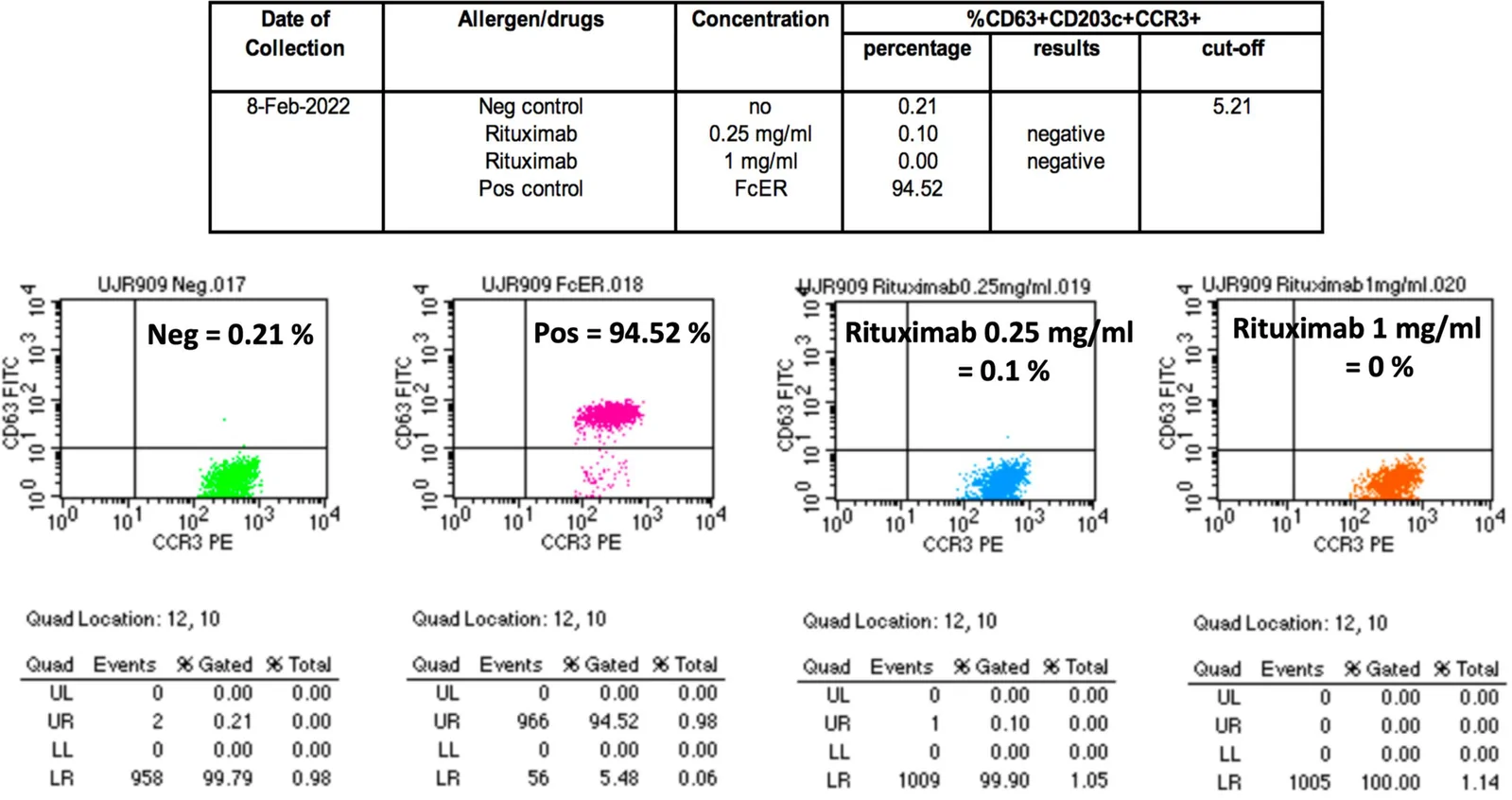
Basophil activation test (BAT) to rituximab.

**Figure 3:**
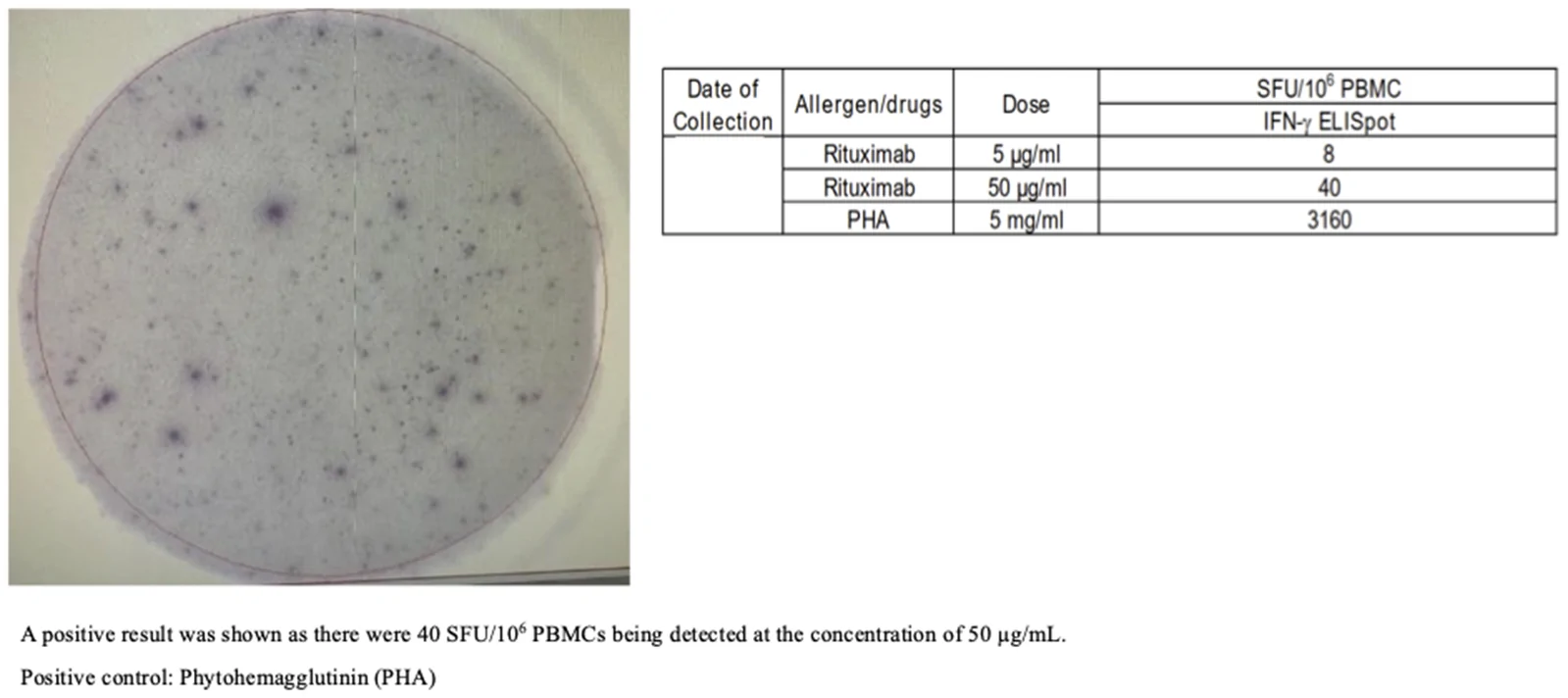
Positive interferon-gamma (IFN-*γ*) enzyme-linked immunospot (ELISpot) assay result in a patient with immediate rituximab hypersensitivity reaction.

Among the 8 patients diagnosed with immediate RTX HSRs, positive RTX skin test results, elevated serum tryptase levels, and positive ADA ELISA results were each observed in 2 of 8 patients. The median interval from the index reaction to skin testing was 6.9 months (IQR, 1.7–13.1 months). BAT results were negative in all evaluated patients, including those with finding suggestive of possible type I reaction as shown in Figure 2. Elevated serum IL-6 levels were detected in 2 patients, whereas a positive IFN-*γ* ELISpot response was identified in 1 patient as shown in Figure 3.

## Discussion

In this prospective pharmacovigilance study, IRRs were the most common clinical phenotype of immediate RTX reactions, occurring in 9 of 17 patients. Consistent with the well-established clinical characteristics of RTX-associated IRRs, all reactions occurred during the first treatment cycle, were mild to moderate in severity (Brown grade I–II), and responded to premedication and/or reduction of the infusion rate. In contrast, RTX HSRs predominantly developed during the third or fourth treatment cycles and were more frequently associated with moderate-to-severe reactions according to Brown's classification (16, 17). These findings suggest that the timing of onset and reaction severity may provide useful clues for distinguishing IRRs from immune-mediated RTX HSRs in routine clinical practice.

Multiple immunologic mechanisms may contribute to RTX HSRs, highlighting the importance of endotype characterization for mechanism-based management (4). In patients who develop reactions beyond the first RTX treatment cycle, further investigation of the underlying mechanism should be considered. Among the patients who underwent endotype evaluation, possible type I and mixed reactions were the most frequently identified classified endotypes, although half of the cases unclassified because of incomplete biomarker data and limitations of current available diagnostic tools. This observation is consistent with previous reports demonstrating that type I reactions are the most common endotype of monoclonal antibody hypersensitivity, followed by mixed reactions (5). Furthermore, ADA positivity has previously been associated with an increased risk of biologic HSRs (4).

Notably, none of the patients in the IRR group demonstrated biomarker evidence of an immune-mediated mechanism. All skin tests, serum tryptase measurements, BAT, IFN-*γ* ELISpot assays, and ADA ELISA results were negative. These findings support the proposed non-immunologic pharmacologic mechanism underlying first-infusion RTX-associated IRRs (17).

Among patients with immediate RTX HSRs who underwent endotype evaluation (*n* = 8), two patients were classified as having possible type I reactions and two as having mixed reactions, whereas four remained unclassified. Isabwe et al. (5) reported type I, cytokine release, mixed, and delayed type IV reactions in 63%, 13%, 21%, and 3% of patients, respectively, in a larger cohort of monoclonal antibody HSRs. The differing endotype distribution observed in our study should be interpreted cautiously, given the small sample size, heterogeneity of underlying diseases, and incomplete biomarker assessment. Notably, half of the patients with immediate RTX HSRs could not be assigned to a specific endotype despite comprehensive evaluation using currently available biomarkers. This finding highlights the limitations of existing diagnostic tools in real-world clinical practice, particularly when acute-phase biomarker sampling cannot be consistently obtained within the optimal time window. Incomplete biomarker data may have contributed to underrecognition of cytokine release syndrome or mixed reactions. Similar challenges have been reported in previous studies, in which a substantial proportion of RTX reactions could not be assigned to a definitive endotype (18). The predominance of reactions during the third and fourth RTX treatment cycles further supports the role of adaptive immune sensitization following repeated antigen exposure (19).

Positive RTX skin test results were identified in 2 of 8 patients with immediate HSRs, and all positive results were detected by IDT rather than SPT. This finding is consistent with previous reports demonstrating the limited sensitivity of SPT and supporting IDT as the preferred *in vivo* method for detecting RTX sensitization (4, 18). Skin testing was performed at least four weeks after the index reaction in accordance with current recommendations to minimize false-negative results related to transient mast cell and basophil refractoriness following acute reactions (4). Elevated serum tryptase levels, assessed using serial sampling and the international consensus equation, were observed in 2 of 8 patients, providing objective evidence of mast cell activation.

BAT results were negative in all patients, including those with biomarker evidence suggestive of type I reactions. Similar findings have been reported in studies of biologic hypersensitivity reactions. Several factors may contribute to reduced BAT sensitivity in RTX HSRs, including differences in activation pathways, the potential involvement of non-IgE-mediated mechanisms, and the effects of underlying immune dysregulation or concomitant immunosuppressive therapy (20). Therefore, negative BAT results should be interpreted cautiously and should not exclude an immune-mediated mechanism.

Elevated serum IL-6 levels were detected in 2 of 8 patients classified as having mixed reactions. Serum IL-6 measurement may be helpful for identifying CRS and may complement serum tryptase in the endotype-based evaluation of monoclonal antibody HSRs (7). However, the diagnostic performance of IL-6 is influenced by the timing of sample collection. In addition, systemic corticosteroid treatment may suppress IL-6 production and result in falsely low levels. Consequently, delayed sampling or corticosteroid administration before blood collection may have contributed to under recognition of CRS-associated reactions in our cohort.

IFN-*γ* ELISpot is a functional assay that measures antigen-specific cellular immune responses and reflects T-cell activation (21). To our knowledge, this is the first study to evaluate IFN-*γ* ELISpot responses in patients with immediate RTX HSRs. A positive IFN-*γ* ELISpot response was identified in 1 of 8 patients and corresponded with ADA positivity. Although the clinical significance of this finding remains unclear, it raises the possibility that cellular immune responses may contribute to selected cases of RTX HSRs. Further studies are required to determine the diagnostic utility and mechanistic relevance of IFN-*γ* ELISpot in biologic hypersensitivity reactions.

Most of patients in our cohort, 15 of 17 patients, received biosimilar rituximab, reflecting institutional prescribing practice. Product switching, primarily driven by annual procurement policies rather than clinical considerations, was documented in 6 of 8 patients with immediate RTX HSRs. Although no clear temporal association between switching and reaction onset was observed, immediate RTX HSRs occurred predominantly during the third and fourth treatment cycles, suggesting that cumulative antigen exposure and adaptive immune sensitization may play a greater role than switching alone. Notably, ADA positivity was detected only in two patients who had undergone product switching, with reactions occurring during the third and fourth cycles after switching. Although this pattern may suggest secondary immune sensitization, a causal relationship could not be established because pre-switch ADA measurements were unavailable.

Current literature indicates that switching between originator biologics and biosimilars is generally not associated with clinically meaningful increases in immunogenicity or safety concerns (22). However, existing studies may be underpowered to detect rare hypersensitivity reactions, particularly with monoclonal antibodies such as rituximab (23). Therefore, while product-related immunogenicity may contribute to ADA formation in selected cases, our data do not support a definitive causal association.

Several limitations should be acknowledged. First, Serum tryptase and IL-6 measurements were unavailable in several cases because acute-phase blood sampling could not always be completed within the predefined sampling window in routine clinical practice. As this study was conducted within a real-world hospital-based pharmacovigilance program, biomarker samples could not always be obtained at the onset of reactions. This incomplete biomarker assessment may have introduced classification bias, particularly by underestimating CRS or mixed endotypes, and may have contributed to the relatively high proportion of unclassified reactions. Second, the sample size was relatively small, limiting the precision of prevalence estimates and endotype classification. Finally, half of the investigated RTX HSRs remained unclassified despite comprehensive biomarker evaluation, suggesting that additional biomarkers may be necessary to better characterize the underlying mechanisms of these reactions. Another important limitation of this study is the heterogeneity of the underlying diseases requiring RTX treatment. Our cohort included patients with diverse conditions, including autoimmune diseases, primary CNS lymphoma, and anti-interferon-*γ* autoantibody-associated disease, which may differ substantially in baseline immune activation, cytokine profiles, prior RTX exposure, concomitant immunosuppressive therapy, and corticosteroid use. These disease-related factors may have influenced both the clinical phenotype of RTX HSRs and the biomarker profiles used for endotype classification, particularly serum IL-6 levels, ADA positivity, BAT responses, and IFN-*γ* ELISpot results. Consequently, the observed endotype distribution may partly reflect disease-specific immunologic differences rather than RTX-related mechanisms alone. Larger studies with more homogeneous patient populations are needed to better define the impact of underlying disease on RTX HSR phenotypes and endotypes.

Despite these limitations, our findings support the utility of integrating clinical phenotypes with biomarker-based investigations to improve the characterization of RTX-induced immediate HSRs. Future multicenter studies with larger cohorts are needed to validate these findings and further refine endotype-based approaches for the diagnosis and management of biologic hypersensitivity reactions.

## Conclusion

Infusion-related reactions were the most common immediate reactions to rituximab and occurred primarily during the first treatment cycle. Immediate rituximab hypersensitivity reactions demonstrated heterogeneous endotypes, with type I and mixed reactions identified among classified cases. Skin testing, anti-drug antibody detection, and serum IL-6 measurement may contribute to endotype classification and warrant further evaluation in larger prospective studies before their routine clinical utility can be established.

## Data Availability

The original contributions presented in the study are included in the article/Supplementary Material, further inquiries can be directed to the corresponding author.
