# Supplementary material for: Clinical phenotypes, endotypes, and biomarker profiles of immediate rituximab hypersensitivity reactions: a prospective pharmacovigilance study
Source: Front Allergy. 2026 Sep 3;7:1903523. doi: 10.3389/falgy.2026.1903523 (PMC13581838; doi:10.3389/falgy.2026.1903523)
Supplement: Supplementary file 1 [file Table1.docx]

Supplement Table S1. Clinical characteristics and potential confounding factors of patients with immediated rituximab hypersensitivity reactions.

| Case No. | Underlying disease | Disease activity at reaction | Prior RTX exposure | Prior biologic exposure | Duration from reaction to skin test (months) | Concomitant immunosuppressive medications (dose/day) | Premedication protocol |
| --- | --- | --- | --- | --- | --- | --- | --- |
| 1 | SLE | A | Y | N | 1.7 | MMF 1,500 mg, Pred 10 mg | MP 100 mg, Diphen 25 mg |
| 2 | Stiff person syndrome, Severe AA, CGVHD | A | Y | N | 19.7 | N | Dexa 5 mg, CPM 10 mg, Diphen 50 mg, Para 1,000 mg |
| 3 | Pemphigus vulgaris | A | Y | N | 1.2 | AZA 50 mg, Pred 20 mg | Dexa 10 mg, Diphen 50 mg, Para 1,000 mg, OMZ 40 mg |
| 4 | Anti-IFN-γ auto-Ab | A | Y | N | 11.9 | N | MP 100 mg, Diphen 25 mg, para 500 mg |
| 5 | Primary CNS Lymphoma | A | Y | N | 12.3 | N | MP 100 mg, CPM 10 mg, OMZ 40 mg |
| 6 | Rheumatoid arthritis | A | Y | Y | 1.5 | MTX 25 mg/wk, HCQ, Leflunomide 20 mg/d | MP 100 mg, CPM 10 mg, Para 1,000 mg |
| 7 | Pemphigus foliaceus | A | Y | N | 15.4 | Pred 10 mg/d | Dexa 10 mg, Diphen 50 mg, Para 1,000 mg, OMZ 40 mg |
| 8 | IgG4-related disease | A | Y | N | 2.0 | AZA 100 mg, Pred 5 mg | MP 100 mg, Diphen 25 mg |

A; active, AA: aplastic anemia, Anti-IFN-γ auto-Ab; anti-interferon-gamma autoantibody, AZA; azathioprine, CGVHD; chronic graft versus host disease, CNS; central nervous system, CPM; chlorpheniramine, Dexa; dexamethasone, Diphen; Diphenhydramine, HCQ; hydroxychloroquine, mg; milligram, MP; methylprednisolone, MTX; methotrexate, N; no, Para; paracetamol, Pred; prednisolone, SLE; systemic lupus erythematosus, Y; yes
